# BMI is Strongly Associated With Hypertension, and Waist Circumference is Strongly Associated With Type 2 Diabetes and Dyslipidemia, in Northern Chinese Adults

**DOI:** 10.2188/jea.JE20110120

**Published:** 2012-07-05

**Authors:** Ren-Nan Feng, Chen Zhao, Cheng Wang, Yu-Cun Niu, Kang Li, Fu-Chuan Guo, Song-Tao Li, Chang-Hao Sun, Ying Li

**Affiliations:** 1 Department of Nutrition and Food Hygiene, School of Public Health, Harbin Medical University, Harbin, P. R. China; 2 Endemic Disease Control Center, Chinese Center for Disease Control and Prevention, Harbin Medical University, Harbin, P. R. China; 3 Department of Biostatistics, School of Public Health, Harbin Medical University, Harbin, P. R. China

**Keywords:** obesity, chronic disease, cut-off value

## Abstract

**Background:**

Obesity is closely associated with chronic diseases such as hypertension, type 2 diabetes mellitus (T2DM), and dyslipidemia. We analyzed the optimal obesity index cut-off values for metabolic syndrome (MetS), and identified the obesity index that is more closely associated with these chronic diseases, in a population of northern Chinese.

**Methods:**

We surveyed 8940 adults (age, 20–74 years) living in northern China for chronic diseases. Receiver operating characteristics (ROC) analysis, relative risk, and multivariate regression were used to develop an appropriate index and optimal cut-off values for MetS and obesity-related chronic diseases.

**Results:**

Waist circumference (WC) and body mass index (BMI) were good markers for MetS, WC was a good marker for T2DM and dyslipidemia, and BMI was a good marker for hypertension. The optimal BMI cut-off value of MetS was 24 kg/m^2^, and the optimal WC cut-offs were 86 cm and 78 cm in men and women, respectively. Relative risk regression models showed that BMI was associated with hypertension, T2DM, and hypertriglyceridemia and a higher prevalence ratio (PR) for hypertension: 2.35 (95% CI, 2.18–2.50). WC was associated with T2DM, hypertension, and hypertriglyceridemia, with PRs of 2.05 (1.63–2.55) for T2DM and 2.47 (2.04–2.85) for hypertriglyceridemia. In multivariate regression models, the standardized regression coefficients (SRCs) of BMI were greater for SBP and DBP, and the SRC of WC was greater for fasting blood glucose, 2-hour postload blood glucose, triglyceride, and total cholesterol.

**Conclusions:**

Our analysis of a population of northern Chinese indicates that the optimal cut-off values for MetS are WCs of 86 cm in men and 78 cm in women and a BMI of 24 kg/m^2^ in both sexes. BMI was strongly associated with hypertension, while WC was strongly associated with T2DM and dyslipidemia.

## INTRODUCTION

The prevalence of obesity has increased throughout the world, including Asia.^1^^,^^2^ It is a crucial risk factor in many chronic diseases such as hypertension,^3^ dyslipidemia,^4^ and type 2 diabetes mellitus (T2DM).^5^ As compared with people in other regions, Asians tend to develop diabetes at lower levels of obesity and younger ages and to develop complications of diabetes earlier.^1^ The obesity epidemic is thus an enormous threat to health care systems in Asia.

Body mass index (BMI), waist circumference (WC), waist-to-height ratio (WHtR), and waist-to-hip ratio (WHpR) have been used as markers of these chronic diseases in adults. However, there is controversy as to which obesity index is more closely related to hypertension, T2DM, and dyslipidemia. In addition, experts disagree on cut-off values for obesity indices. Recent studies of Japanese and Koreans proposed WC cut-off values that were optimal for identifying at least 2 components of metabolic syndrome (MetS) in those populations.^6^^–^^10^ Furthermore, evidence from some studies indicates that BMI is better than WC as an index of metabolic risk.^11^^–^^13^ However, results from a study of Japanese women suggested that BMI was a better index of overall and abdominal fat in that population.^14^ The International Diabetes Federation (IDF) has strongly recommended that more-extensive investigations be performed to determine optimal cut-off values for clinical practice in different ethnic regions.^15^

The InterASIA study indicated that 64 million Chinese adults aged 35 to 74 years had MetS as defined by Adult Treatment Panel III (ATP III) criteria and that the prevalences of MetS and overweight were higher in northern China than in southern China.^16^ The regional prevalences of hypertension and diabetes were also reported to be higher in northern China.^17^^,^^18^ Differences in diet and physical activity between northern and southern China might contribute to these regional differences in the distribution of chronic disease.^16^ More importantly, the population in northern China was reported to be taller, heavier, and to have higher BMI and triglyceride levels as compared with the southern population,^19^ which suggests that anthropometric cut-off values for these populations require clarification.

Because of the differences between northern and southern Chinese populations and because existing data on anthropometric cut-off values were all reported from studies of southern China, the principal objectives of this study were to identify region-specific optimal anthropometric cut-off values for MetS and develop anthropometric indices that are more closely associated with hypertension, T2DM, and dyslipidemia in a large population of northern Chinese.

## METHODS

### Participants

A total of 3960 men and 4980 women aged 20 to 74 years underwent health examinations in 2008 as part of the Harbin Health Study.^20^ Data were manually collected by the Centers for Disease Control and Prevention and Public Health School in Harbin, the biggest city in northern China. We analyzed data from 8940 residents of 5 different districts in Harbin city as a representative population of northern China. The participants in each of the 5 districts were randomly sampled from 3 communities and matched with financial status. Residents with self-reported cancer or cardiovascular disease and pregnant women were excluded from the analyses. The health examination and measurements were conducted in community clinics by physicians, public health nurses, and medical technologists.

All participants underwent a 75-g oral glucose tolerance test (OGTT). Blood samples were drawn for subsequent laboratory analysis. Data on educational status, physical activity level (PAL), dietary intakes, smoking and drinking status, and medical history were collected using face-to-face questionnaires answered by the participants, as described in our previous study.^21^ Participants who reported current alcohol drinking or smoking (at least once per month) were defined as drinkers or smokers. A participant who had received drug therapy for dyslipidemia, hypertension, or hyperglycemia was recorded as having the respective risk factor regardless of laboratory data. This study was approved by the ethics committee of Harbin Medical University. Written informed consent was obtained from all participants.

### Anthropometric evaluation

Anthropometric indices were measured by well-trained examiners, with participants wearing light, thin clothing and no shoes. Body weight and height were measured to the nearest 0.1 kg and 0.1 cm, respectively. Blood pressure was measured 3 times with a standard mercury sphygmomanometer and appropriately sized adult cuffs (standard, adult large, or thigh cuff) on the right arm of each subject after a 10-minute rest in a sitting position, and the mean values were used for analysis. BMI was calculated as weight (kg) divided by the square of the height (meters). WC was measured midway between the lowest rib and the iliac crest with a flexible anthropometric tape on the horizontal plane with the participant in standing position. Hip circumference was measured over thin clothing at the point of maximum circumference of the buttocks. Both circumferences were measured to the nearest 0.1 cm. The mean of the 3 closest readings was used in subsequent statistical analysis. WHtR was calculated as waist circumference (cm) divided by the height (cm); WHpR was calculated as waist circumference (cm) divided by hip circumference (cm).

### Laboratory analysis

Blood samples were collected after on overnight fast and OGTT testing (without any form of medication) and were immediately centrifuged at 2500 × g for 15 minutes to obtain serum, which was immediately cooled, stored at −80°C, and thawed only once, for measurement of serum concentration of fasting blood glucose (FBG), 2-hour postload blood glucose (2h-PG), total cholesterol (TC), high-density lipoprotein cholesterol (HDL-C), and triglycerides (TG). Blood glucose was measured quantitatively by the glucose oxidase method. Serum TC, HDL-C, and TG were assessed with standard enzymatic methods. All samples were analyzed with an auto-analyzer (MOL-300 Auto-analyzer, China).

### Definitions of multiple metabolic risks, hypertension, T2DM, and dyslipidemia

Participants with multiple metabolic risks were defined as those with 2 or more of the following 4 risk factors from IDF criteria: high TG (TG >1.7 mmol/L [150 mg/dL]); low HDL-C (<1.03 mmol/L [40 mg/dL] in men and <1.29 mmol/L [40 mg/dL] in women); high blood pressure (systolic blood pressure [SBP] ≥130 mm Hg or diastolic blood pressure [DBP] ≥85 mm Hg); and hyperglycemia (FBG level ≥5.6 mmol/L [100 mg/dL] or 2h-PG level ≥7.8 mmol/L [140 mg/dL]) or previously diagnosed T2DM. Hypertension was defined as SBP of 140 mm Hg or higher or DBP of 90 mm Hg or higher. T2DM was defined as FBG level of 7.0 mmol/L (125 mg/dL) or higher or 2h-PG level of 11.1 mmol/L (200 mg/dL) or higher. Hypertriglyceridemia was defined as TG greater than 2.26 mmol/L (200 mg/dL). Hypercholesterolemia was defined as TC greater than 6.22 mmol/L (240 mg/dL).

### Statistical analysis

Statistical analysis was performed using SPSS (version 13.01S; Beijing Stats Data Mining Co. Ltd, Beijing, China) and SAS (version 9.1; SAS Institute, Inc., Cary, NC, USA). Results are presented as mean ± SD. The chi-square test was used to test variation in frequency. The *t* test was used to assess differences in the means of continuous variables.

Receiver operating characteristics (ROC) analysis was used to identify optimal cut-off values for the anthropometric index, with the maximized Youden index (sensitivity plus specificity − 1), to identify multiple risk factors (≥2 components of high TG, high blood pressure, hyperglycemia, and low HDL-C, according to IDF criteria). Furthermore, area under the ROC curve (AUC) was calculated to compare the effectiveness of different anthropometric indices.

Relative risk regression was performed over logistic regression. Because the prevalence of chronic diseases in our study was greater than 10%, odds ratios (ORs) estimated from cross-sectional data overestimate prevalence ratios (PRs). The PRs and 95% CIs of each disease for a high BMI and WC were calculated from the regression model y = exp(X^T^β). The exponentiated parameter β was interpreted as the PR. We assumed Gaussian error and used robust standard error estimates. RPs can be derived from binomial regression models fitted with PROC GENMOD in SAS.^22^

Multivariate linear regression analyses were performed using SBP, DBP, FBG, 2-hPG, TG, TC, and HDL-C as dependent variables and age, BMI, WC, total energy intake energy, and PAL as independent variables. All *P* values were 2-tailed, and a *P* value less than 0.05 was considered to indicate statistical significance.

## RESULTS

### Characteristics of the participants

The characteristics of the male and female participants (3960 men and 4980 women) are shown in Table 1 . As compared with women, men had a significantly lower mean age, WHtR, HDL-C, and rate of dyslipidemia, and significantly higher mean BMI, WC, WHpR, SBP, DBP, FPG, TG, and rates of smoking, drinking, T2DM, and hypertension. Mean 2h-PG values were similar in both sexes.

**Table 1.** Characteristics of participants

Characteristics	Men	Women
*n*	3960	4980
Age (years)	49.40 ± 12.93	50.53 ± 12.00
Education (%)		
≤9 years	36.8	46.5*
10–12 years	30.6	32.1
>12 years	32.6	21.4*
Smoker (%)	45.9	6*
Alcohol drinker (%)	60.1	18.1*
Energy (Kcal)	2242.79 ± 849.26	1745.20 ± 661.43*
PAL	1.43 ± 0.44	1.52 ± 0.46
BMI (kg/m^2^)	25.84 ± 3.70	24.93 ± 3.79*
Waist (cm)	89.35 ± 10.15	81.88 ± 10.06*
WHpR	0.89 ± 0.07	0.84 ± 0.07
WHtR	0.59 ± 0.04	0.62 ± 0.05
SBP (mm Hg)	135.67 ± 20.59	131.12 ± 23.10*
DBP (mm Hg)	83.02 ± 11.61	78.09 ± 11.05*
FBG (mmol/L)	5.18 ± 2.35	5.02 ± 1.66
2h-PG (mmol/L)	6.85 ± 4.17	6.69 ± 3.84
TC (mmol/L)	4.84 ± 0.97	4.88 ± 1.01
TG (mmol/L)	2.19 ± 2.24	1.65 ± 1.32*
HDL-C (mmol/L)	1.25 ± 0.35	1.29 ± 0.34
T2DM or antidiabetic medication (%)	9.3/69.64	8.4*/88.15*
Hypertension or antihypertensive medication (%)	38.4/49.53	29.1*/64.59*
Dyslipidemia or lipid-lowering medication (%)	30.8/30.12	35.6*/35.25*

Data are mean ± SD.

Abbreviations: PAL, physical activity level; BMI, body mass index; WHpR, waist-to-hip ratio; WHtR, waist-to-height ratio; SBP, systolic blood pressure; DBP, diastolic blood pressure; FBG, fasting blood glucose; 2h-PG, 2-h postload blood glucose; TC, total cholesterol; TG, triglycerides; HDL-C, high-density lipoprotein cholesterol; T2DM, type 2 diabetes mellitus.

*P < 0.05, compared with men.

### Optimal obesity indices and corresponding cut-off values for multiple metabolic risks and obesity-related chronic diseases

Table 2 shows the ROC curves for WC, BMI, WHtR, and WHpR used to identify MetS (multiple metabolic risk = ≥2 components) for men and women, according to IDF criteria. The AUCs for WC, BMI, WHpR, and WHtR were 0.78 (95% CI: 0.76–0.79), 0.77 (0.75–0.79), 0.66 (0.69–0.88), 0.64 (0.62–0.66), respectively, for men and 0.75 (0.70–0.76), 0.73 (0.69–0.75), 0.69 (0.67–0.71), and 0.68 (0.67–0.70) for women. WC had the largest AUC value. However, the AUC for BMI was similar to that for WC. A WC of 86 cm for men and 78 cm for women appeared to be the optimal cut-off values for multiple metabolic risk, and 24 kg/m^2^ was the optimal cut-off value for BMI in both sexes. The sensitivity and specificity was 74.45% and 62.63%, respectively, in men and 79.12% and 62.5% in women when using our proposed WC cut-off values. For the proposed BMI cut-off value, the sensitivity and specificity were 75.03% and 60.65%, respectively, in men and 71.70% and 69.60% in women.

**Table 2.** Sensitivity, specificity, and area under the curve (AUC) for cut-off values of anthropometric indices to identify ≥2 metabolic risks

Sex	Index	Cut-off value	Sensitivity (%)	Specificity (%)	Youden index	AUC (95% CI)
Men	WC	86.00	74.45	62.63	37.01	0.78 (0.76–0.79)
	BMI	24.63	75.03	60.65	35.68	0.77 (0.75–0.79)
	WHpR	0.88	71.85	51.11	22.96	0.66 (0.69–0.88)
	WHtR	0.57	75.10	46.30	21.40	0.64 (0.62–0.66)
Women	WC	78.00	79.12	62.50	41.62	0.75 (0.70–0.76)
	BMI	24.2	71.70	69.60	41.30	0.73 (0.69–0.75)
	WHpR	0.83	73.54	56.76	30.30	0.69 (0.67–0.71)
	WHtR	0.61	67.71	62.88	30.58	0.68 (0.67–0.70)

Abbreviations: BMI, body mass index; WC, waist circumference; WHpR, waist-to-hip ratio; WHtR, waist-to-height ratio.

The AUC for WC was greater for T2DM and dyslipidemia, and the AUC for BMI was greater for hypertension among the whole population and individuals with a BMI <30 kg/m^2^ (data not shown). Table 3 shows that the WC cut-off values for T2DM were 87.05 cm and 81.95 cm in men and women, respectively, that the WC cut-off values for dyslipidemia were 88.05 cm and 81.05 cm, and that the BMI cut-off values for hypertension were 24.05 kg/m^2^ and 24.40 kg/m^2^, which were lower or similar to the cut-off values for MetS. Similar results were obtained in subjects with a BMI less than 30 kg/m^2^ (Table S1, see SUPPORTING INFORMATION).

**Table 3.** Sensitivity, specificity, and area under the curve (AUC) for cut-off values of anthropometric indices to identify obesity-related chronic diseases

Sex	Disease	Optimal index	Cut-off	Sensitivity (%)	Specificity (%)	Youden index	AUC (95% CI)
Men	T2DM	WC	87.05	65.28	51.13	16.41	0.60 (0.57–0.63)
	Dyslipidemia	WC	88.05	70.18	58.82	29.00	0.56 (0.54–0.58)
	Hypertension	BMI	24.05	74.68	58.62	33.30	0.58 (0.56–0.61)
Women	T2DM	WC	81.95	75.46	48.14	23.60	0.63 (0.60–0.66)
	Dyslipidemia	WC	81.05	63.99	59.12	23.11	0.58 (0.57–0.60)
	Hypertension	BMI	24.40	71.49	63.32	34.81	0.66 (0.64–0.67)

Abbreviations: BMI, body mass index; T2DM, type 2 diabetes mellitus; WC, waist circumference.

When stratified by age (<50 vs ≥50 years, Table S2, see SUPPORTING INFORMATION), the cut-off values for multiple metabolic risk were similar for subjects younger than 50 years and the whole population; however, among older subjects, the cut-off values for multiple metabolic risk, T2DM, and dyslipidemia were higher than in younger subjects, whereas the cut-off value for hypertension was similar.

### Association between WC, BMI, and chronic diseases and parameters

Table 4 shows the PRs for chronic health conditions, with obesity defined by the proposed WC and BMI cut-off values in the same model. PRs for a high WC (≥86 in men; ≥78 cm in women) were greater for T2DM and hypertriglyceridemia, and the PR for a BMI of 24 kg/m^2^ or higher was greater for hypertension.

**Table 4.** Relative risk regressions using T2DM, hypertension, hypertriglyceridemia, hypercholesterolemia, and low HDL-C as dependent variables

	Men		Women		Total	
		
	BMI^a^	WC^a^	BMI^a^	WC^a^	BMI^b^	WC^b^
T2DM	1.18 (0.75–1.61)	1.98 (1.38–2.68)	1.43 (1.13–1.73)	2.07 (1.27–2.79)	1.37 (1.13–1.61)	2.05 (1.63–2.55)
Hypertension	2.48 (1.95–2.85)	1.50 (1.01–1.88)	2.33 (1.99–2.69)	1.79 (1.61–1.97)	2.35 (2.18–2.50)	1.79 (1.52–2.07)
Hypertriglyceridemia	1.61 (1.20–2.00)	2.46 (2.02–2.89)	1.68 (1.35–2.07)	2.55 (2.14–2.88)	1.66 (1.40–1.95)	2.47 (2.04–2.85)
Hypercholesterolemia	1.40 (0.89–2.15)	1.49 (1.00–2.18)	1.00 (0.78–1.35)	1.22 (1.01–1.66)	1.10 (0.91–1.29)	1.38 (1.08–1.71)
Low HDL-C	1.01 (0.78–1.27)	1.15 (0.90–1.41)	1.01 (0.90–1.26)	1.10 (0.91–1.30)	1.03 (0.93–1.18)	1.14 (1.01–1.26)

^a^Prevalence ratios of diseases for a BMI of 24 and a WC of 86 cm in men or 78 cm in women, after adjustment for age, education, smoking, drinking, total energy intake, medication status, and physical activity level in the same model.

^b^Additionally adjusted for sex.

Table 5 shows the results of multiple linear regressions using SBP, DBP, FBG, 2h-PG, TG, TC, and HDL-C as dependent variables and age, BMI, WC, total energy intake, medication status, and PAL as independent variables in men, women, and the overall population. The standardized regression coefficients (SRCs) of BMI were greater for SBP and DBP, and the SRCs of WC were greater for TG, TC, FBG, and 2h-PG. These findings indicate that BMI was more strongly associated with hypertension and that WC was more strongly associated with T2DM and dyslipidemia.

**Table 5.** Multivariate linear regressions using SBP, DBP, FPG, 2h-PG, TG, TC, and HDL-C as dependent variables

	SBP	DBP	FPG	2h-PG	TG	TC	HDL-C
Men							
BMI^a^	0.210**	0.197**	−0.014	0.002	0.102**	0.06*	0.004
WC^a^	0.058*	0.127**	0.152**	0.168**	0.137**	0.111**	−0.084*
BMI^b^	0.206**	0.199**	−0.013	0.006	0.106**	0.073*	0.003
WC^b^	0.076*	0.129**	0.152**	0.162**	0.129**	0.103**	−0.09*
Women							
BMI^a^	0.197**	0.210**	0.024	0.038	0.068**	−0.012	−0.038
WC^a^	0.148**	0.105**	0.156**	0.19**	0.153**	0.122**	−0.043
BMI^b^	0.185**	0.213**	0.026	0.039	0.070**	−0.013	−0.035
WC^b^	0.138**	0.103**	0.152**	0.176**	0.153**	0.117**	−0.042
Total							
BMI^c^	1.95**	0.201**	0.009	0.026	0.079**	0.011	−0.023
WC^c^	1.29**	0.121**	0.161**	0.181**	0.171**	0.148**	−0.063**
BMI^d^	1.90**	0.203**	0.01	0.028	0.082**	0.012	−0.025
WC^d^	1.24**	0.111**	0.159**	0.179**	0.167**	0.141**	−0.062**

^a^Standardized regression coefficients (SRCs) were adjusted for age.

^b^Adjusted for age, total energy intake, medication status, and physical activity level.

^c^Adjusted for age and sex.

^d^Adjusted for age, sex, total energy intake, and physical activity level.

Abbreviations: BMI, body mass index; WC, waist circumference; SBP, systolic blood pressure; DBP, diastolic blood pressure; FBG, fasting blood glucose; 2h-PG, 2-h postload blood glucose; TC, total cholesterol; TG, triglycerides; HDL-C, high-density lipoprotein cholesterol *P < 0.05; **P < 0.001.

## DISCUSSION

Obesity prevalence has dramatically increased in China recent years,^16^ and obesity has been shown to be a risk factor for hypertension,^3^ dyslipidemia,^4^ and T2DM.^5^ However, it is not clear which anthropometric indices of obesity, eg, BMI, WC, WHpR, and WHtR, are more closely associated with hypertension, T2DM, and dyslipidemia. The Decoda Study Group reported that WHpR was more strongly associated than BMI with diabetes, but both these indicators were also reported to be associated with hypertension in Asians.^23^ However, Oda and Kawai reported that BMI was more strongly associated than WC with hypertension in apparently healthy Japanese men and women.^24^ Among risk factors for T2DM, BMI is regarded as the most important index,^25^ but Lorenzo et al concluded that WC was optimal for predicting T2DM.^26^ The data suggest that associations between anthropometric indices and obesity-related chronic diseases differ by population.

In addition, cut-off values for obesity indices have been challenged in many Asian countries. It has been reported that the WC specified in the IDF criteria for MetS may be greater for some Asian populations^8^ and that its effectiveness may not be better than that of BMI and other anthropometric indices.^14^ It is of great interest to determine sensitive indices and accurate cut-off values for metabolic risks and to identify the indices that are more sensitive to hypertension, dyslipidemia, and T2DM. In our large sample (*n* = 8940), the optimal WC cut-off values were 86 cm in men and 78 cm in women, and the optimal BMI cut-off value was 24 kg/m^2^ in both sexes, for obesity-related multiple metabolic risks (MetS). In addition, we were the first to find that WC was more strongly associated with T2DM and dyslipidemia, and that BMI was more strongly associated with hypertension, in this population of northern China, although WC and BMI were significantly related with each other (Figure S1, see SUPPORTING INFORMATION).

Our proposed WC and BMI cut-off values are lower than those reported by Bao et al^27^ and Zhou et al,^28^ which might be due to differences in lifestyle and environment factors between northern and southern China. For example, in the cold climate of northern China people tend to be less physically activity, thereby increasing the risks of chronic diseases and severe metabolic abnormalities.^29^^,^^30^ However, the age range we sampled (mean, 51; range, 20–74) was much wider than that of a previous study (mean, 54; range, 47–62).^27^ Because WC is greater in older populations, this might be a reason for the inconsistent findings. To examine this possibility, we stratified data by age (<50 and ≥50 years). Individuals younger than 50 years had cut-off values similar to those of the overall population; however, the cut-off values for multiple metabolic risk among the older group (WC: 88 cm for men, 81 cm for women; BMI: 25 kg/m^2^ for men and women) were greater than those for younger adults.

Our results were in close agreement with those of a previous study, which reported similar results (WC: 85 cm in men and 80 cm in women)^28^; however, our study also found that a BMI of 24 kg/m^2^ or higher should be considered an equivalent risk for chronic diseases, because the AUC predicting multiple metabolic risks was similar for BMI and WC. In addition, these measures were strongly associated with chronic diseases, which was supported by a cohort study of a Hong Kong population, which defined a BMI of 23 kg/m^2^ or greater as pre-obesity.^31^ However, they defined a large range of WC as high WC, ie, 84 to 90 cm in men and 74 to 80 cm in women, which is difficult to apply clinically.

There are several reasons why BMI is strongly associated with hypertension while WC is strongly independently associated with T2DM and hyperlipemia in northern Chinese. First, BMI represents overall body weight but is unable to distinguish between excess adipose tissue and high muscle mass. WC is a simple measure of abdominal obesity and might better reflect accumulation of intra-abdominal fat. However, reports indicate that Chinese have a higher body fat content and are at a higher risk for diabetes, high blood pressure, and heart disease than individuals with the same BMI in other populations.^1^ Visceral fat in abdominal obesity is the main source of free fatty acids and inflammatory cytokines and could lead to insulin resistance and T2DM, which explains why WC was more strongly associated with T2DM and dyslipidemia in our findings. Therefore, to prevent and diagnose T2DM and dyslipidemia, WC should be carefully assessed, even when BMI is not high. Second, it seems that weight is more strongly associated than abdominal obesity with hypertension and blood pressure, because an increase in body weight (BMI) increases body fluid volume, peripheral resistance (eg, hyperinsulinemia, cell membrane alteration, and hyperactivity of the renin-angiotensin system leading to functional constriction and structural hypertrophy), and cardiac output.^32^ The positive association between WC and hypertension could be the result of excess visceral fat that leads to high levels of leptin and inflammatory cytokines, insulin resistance, and lipid disorders.

Several additional points warrant discussion. First, the findings of this cross-sectional study are not conclusive evidence of a causal relation of WC and BMI with cardiovascular disease. Thus, we must be cautious in interpreting the present results, and further cohort studies are needed to clarify our findings. Second, as the data were from a northern Chinese population, our proposed cut-off values for the indices are only valid for this population. Third, MetS was associated with increases in cardiovascular disease risk and cardiovascular mortality rate in Chinese.^33^^,^^34^ However, no preventive measures have been found to be effective among Chinese with MetS. Early diagnosis and management of metabolic disorders and chronic diseases is essential; thus, our results might provide valuable suggestions for screening and preventing chronic diseases.

In conclusion, in diagnosing MetS, we recommend optimal WC cut-off values of 86 cm in men and 78 cm in women and a BMI of 24 kg/m^2^ in both sexes. BMI was strongly associated with hypertension, and WC was strongly associated with T2DM and dyslipidemia in a population of northern Chinese.

## SUPPORTING INFORMATION

Supplementary materials are available on the journal’s website at http://dx.doi.org/10.2188/jea.JE20110120.

eTables.

eFigures.
