# Supplementary material for: BMI is Strongly Associated With Hypertension, and Waist Circumference is Strongly Associated With Type 2 Diabetes and Dyslipidemia, in Northern Chinese Adults
Source: J Epidemiol. 2012 Jul 5;22(4):317–23. doi: 10.2188/jea.JE20110120 (PMC3798650; doi:10.2188/jea.JE20110120)
Supplement: eTables. [file je-22-317-s001.pdf]

Table S1. Sensitivity, specificity, and area under the curve (AUC) for cut-off values of anthropometric indices to identify obesity-related chronic diseases in participants with BMI <30 kg/m^2

Sex	Disease	Optimal index	Cut-off	Sensitivity (%)	Specificity (%)	Youden index	AUC (95% CI)
Men	≥ 2 metabolic risks	WC	87.05	70.03	66.31	36.34	0.77(0.76,0.79)
	≥ 2 metabolic risks	BMI	24.63	70.69	64.33	35.02	0.76(0.75,0.78)
	T2DM	WC	87.05	72.61	50.44	23.05	0.64(0.61,0.66)
	Dyslipidemia	WC	88.95	63.61	60.21	23.82	0.62(0.60,0.64)
	Hypertension	BMI	24.59	70.48	58.58	29.06	0.66(0.64,0.68)
Women	≥ 2 metabolic risks	WC	78.45	73.72	62.91	36.63	0.74(0.73,0.78)
	≥ 2 metabolic risks	BMI	24.20	75.47	61.29	36.76	0.70(0.69,0.73)
	T2DM	WC	83.85	61.34	67.15	28.49	0.69(0.67,0.71)
	Dyslipidemia	WC	81.35	60.52	61.47	21.99	0.67(0.66,0.69)
	Hypertension	BMI	24.58	71.09	63.13	34.22	0.69(0.67,0.70)

Abbreviations: BMI, body mass index; WC, waist circumference; T2DM, type 2 diabetes mellitus.

Table S2. Sensitivity, specificity, and area under the curve (AUC) for cut-off values of anthropometric indices to identify obesity-related chronic diseases in participants aged ≥50 and <50 years

Sex	Disease	Optimal index	Cut-off	Sensitivity (%)	Specificity (%)	Youden index	AUC (95% CI)
Men	<50 years						
	≥ 2 metabolic risks	WC	86.45	69.72	63.33	33.05	0.70(0.68,0.73)
	≥ 2 metabolic risks	BMI	24.72	76.21	63.62	39.83	0.69(0.66,0.71)
	T2DM	WC	87.05	68.74	58.91	27.65	0.64(0.60.0.69)
	Dyslipidemia	WC	88.65	63.86	63.80	27.66	0.63(0.60,0.66)
	Hypertension	BMI	24.86	64.64	62.16	26.80	0.67(0.64,0.70)
	>=50 years						
	≥ 2 metabolic risks	WC	88.70	73.67	59.41	33.08	0.69(0.65,0.69)
	≥ 2 metabolic risks	BMI	25.71	69.77	63.52	33.29	0.68(0.65,0.68)
	T2DM	WC	89.05	77.23	44.25	21.48	0.63(0.59,0.66)
	Dyslipidemia	WC	89.95	63.87	62.87	26.74	0.60(0.58,0.64)
	Hypertension	BMI	24.60	73.85	57.20	31.05	0.64(0.62,0.67)
Women	< 50 years						
	≥ 2 metabolic risks	WC	76.85	65.28	64.92	30.20	0.70(0.67,0.72)
	≥ 2 metabolic risks	BMI	24.12	66.14	67.25	33.39	0.70(0.66,0.71)
	T2DM	WC	81.35	64.43	66.89	31.32	0.68(0.64,0.72)
	Dyslipidemia	WC	81.05	60.45	67.56	28.15	0.54(0.52,0.57)
	Hypertension	BMI	24.30	72.75	67.53	40.31	0.74(0.72,0.77)
	>=50 years						
	≥ 2 metabolic risks	WC	81.15	69.46	65.97	35.43	0.68(0.66,0.69)
	≥ 2 metabolic risks	BMI	25.14	63.75	69.23	32.98	0.67(0.65,0.69)
	T2DM	WC	83.85	70.34	50.09	20.43	0.64(0.62,0.67)
	Dyslipidemia	WC	83.15	59.76	62.08	21.84	0.57(0.55.0.59)
	Hypertension	BMI	24.82	69.64	69.76	39.40	0.67(0.65,0.69)

BMI, body mass index; WC, waist circumference; T2DM, type 2 diabetes mellitus.
