# Supplementary material for: BMI is Strongly Associated With Hypertension, and Waist Circumference is Strongly Associated With Type 2 Diabetes and Dyslipidemia, in Northern Chinese Adults
Source: J Epidemiol. 2012 Jul 5;22(4):317–23. doi: 10.2188/jea.JE20110120 (PMC3798650; doi:10.2188/jea.JE20110120)
Supplement: eFigures. [file je-22-317-s002.pdf]

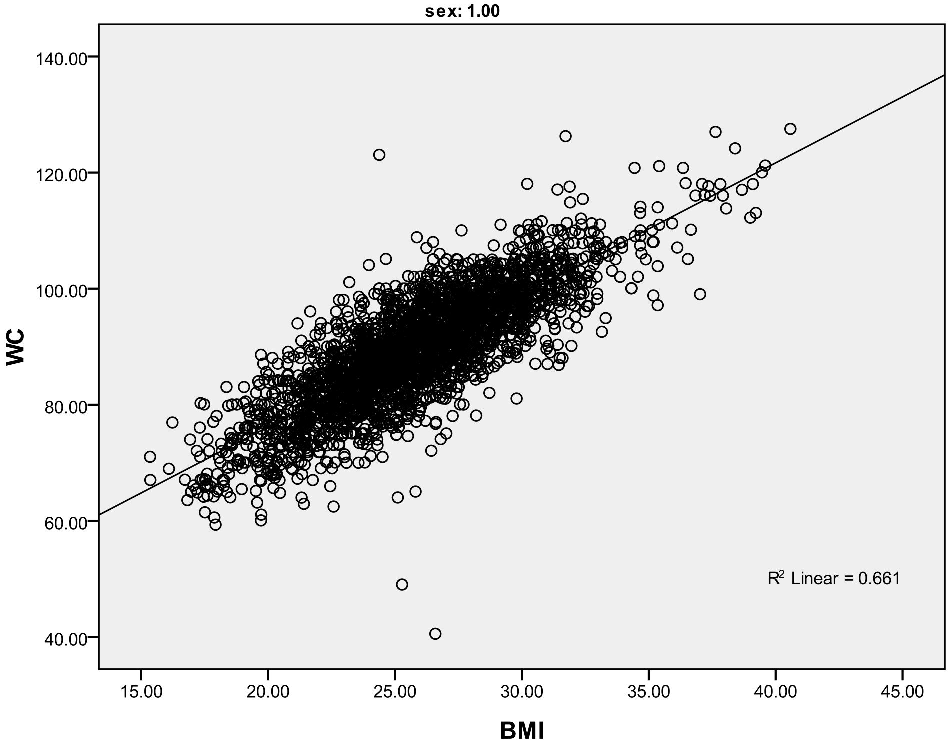

sex: 1.00
140.00
120.00
100.00
80.00
60.00
40.00
WC
15.00
20.00
25.00
30.00
35.00
40.00
45.00
BMI
R² Linear = 0.661

A

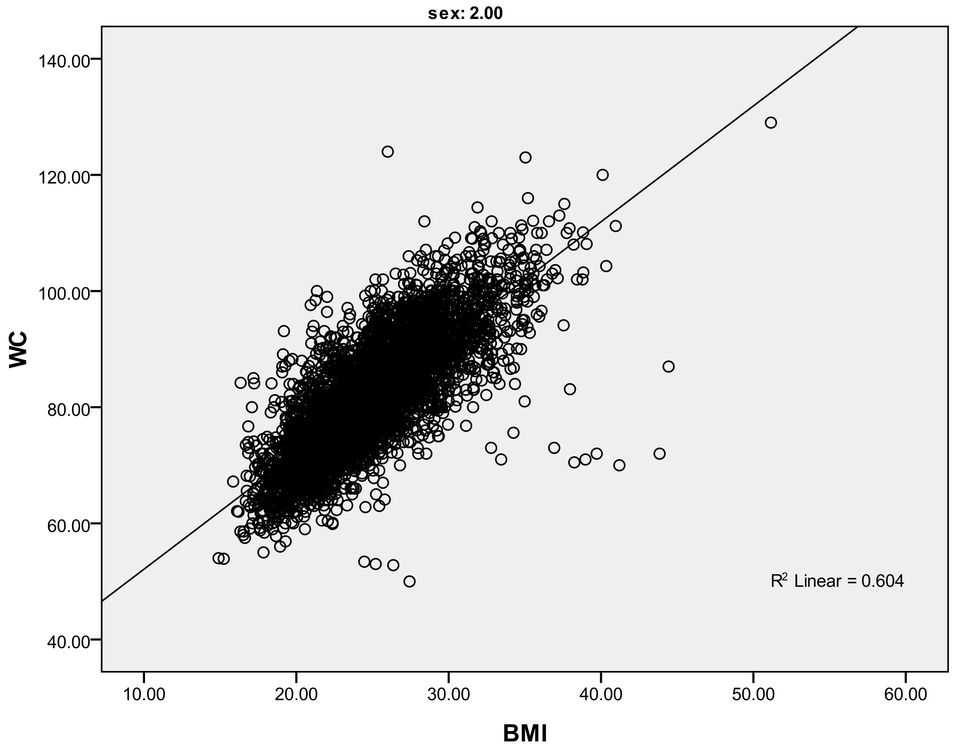

B

Figure S1. Correlation between waist circumference (WC) and body mass index (BMI) (A: men, *r*=0.813, *P*<0.001; B: women, *r*=0.777, *P*<0.001).
